# Real-world Trends, Rural-urban Differences, and Socioeconomic Disparities in Utilization of Narrow versus Broad Next-generation Sequencing Panels

**DOI:** 10.1158/2767-9764.CRC-23-0190

**Published:** 2024-02-05

**Authors:** Yiqing Zhao, Anastasios Dimou, Zachary C. Fogarty, Jun Jiang, Hongfang Liu, William B. Wong, Chen Wang

**Affiliations:** 1 Department of Artificial Intelligence and Informatics, Mayo Clinic, Rochester, Minnesota.; 2 Department of Oncology, Mayo Clinic, Rochester, Minnesota.; 3 Department of Quantitative Health Sciences, Mayo Clinic, Rochester, Minnesota.; 4 Genentech, South San Francisco, California.

## Abstract

Advances in genetic technology have led to the increasing use of genomic panels in precision oncology practice, with panels ranging from a couple to hundreds of genes. However, the clinical utilization and utility of oncology genomic panels, especially among vulnerable populations, is unclear. We examined the association of panel size with socioeconomic status and clinical trial matching. We retrospectively identified 9,886 eligible adult subjects in the Mayo Clinic Health System who underwent genomic testing between January 1, 2016 and June 30, 2020. Patient data were retrieved from structured and unstructured data sources of institutional collections, including cancer registries, clinical data warehouses, and clinical notes. Socioeconomic surrogates were approximated using the Area Deprivation Index (ADI) corresponding to primary residence addresses. Logistic regression was performed to analyze relationships between ADI or rural/urban status and (i) use of genomic test by panel size; (ii) clinical trial matching status. Compared with patients from the most affluent areas, patients had a lower odds of receiving a panel test (vs. a single-gene test) if from areas of higher socioeconomic deprivation [OR (95% confidence interval (CI): 0.71 (0.61–0.83), *P* < 0.01] or a rural area [OR (95% CI): 0.85 (0.76–0.96), *P* < 0.01]. Patients in areas of higher socioeconomic deprivation were less likely to be matched to clinical trials if receiving medium panel tests [(OR) (95% CI): 0.69 (0.49–0.97), *P* = 0.03]; however, there was no difference among patients receiving large panel tests (*P* > 0.05) and rural patients were almost 2x greater odds of being matched if receiving a large panel test [(OR) (95% CI): 1.76 (1.21–2.55), *P* < 0.01].

**Significance:**

We identified socioeconomic and rurality disparities in the use of genomic tests and trial matching by panel size, which may have implications for equal access to targeted therapies. The lack of association between large panel tests and clinical trial matching by socioeconomic status, suggests a potential health equity impact, while removing barriers in access to large panels for rural patients may improve access to trials. However, further research is needed.

## Introduction

Biomarker testing in oncology has made significant advancements in recent years. In particular, advancements in next-generation sequencing (NGS) technologies, for example, faster turnaround time and lower sequencing costs, have contributed to a much wider embracement of Precision Oncology (1) in oncology clinical practice. The potential of Precision Oncology is to enable oncology practitioners to incorporate individual patients’ genomic and clinical characteristics into the clinical decision-making process and subsequently avoid side effects from ineffective or toxic therapies, reducing health care costs and disparity while improving patient outcomes (2–5).

There have been several multi-gene panel tests (MGPT), varying in size, that have become available for oncologists to use. A study examining real-world utilization of MGPTs in Medicare Advantage patients found that large panels have increased in utilization over time (6). In addition, some studies have found larger panels may provide a larger gain in actionability with FDA-approved or experimental drugs via clinical trials (7, 8). Despite the higher upfront costs, larger panels may also reduce total cancer care costs (9). Conversely, a potential limitation of MGPT may be longer turnaround times, as delays in test results may lead to initiation of chemotherapy (10, 11) and delay targeted therapy initiation. Wong and colleagues (10) and Illei and colleagues (12) found longer turnaround times compared with single-gene testing, although the mean times of 13 days were found to be within the guidelines of 2 weeks. Differences in turnaround times between different-sized panel tests, however, are less clear. Finally, there is a lack of consensus on the choice of optimal panel size, and data on real-world utilization by panel size are limited.

Despite the lack of clarity on optimal panel size, the use of MGPTs and targeted therapy (Precision Oncology) in clinical practice has increased (13) and has contributed to decreases in mortality in certain cancer types (14). Although MGPT use has increased over the years, socioeconomic and geographic disparities in access to MGPT have persisted. Previous research has shown socioeconomic status (SES) to be associated with the use of biomarker testing, as well as MGPTs specifically. Gross and colleagues found that Medicaid beneficiaries were less likely to receive biomarker testing and targeted therapies compared with commercially insured patients (15). In addition, the gap in the use of MGPT testing between Medicaid and Medicare has been shown to persist despite the implementation of the NGS Medicare national coverage determination (16). Geographic differences in genomic testing resources may be a contributor to differences in access to biomarker testing, with fewer practices in rural areas (vs. urban practices) having on-site pathology, genetic counselors, protocols for genomic tests, and molecular tumor boards (17). While disparities in the use of MGPT have been documented, less is known about whether these disparities exist between different-sized genomic panel tests as well. Disparities in panel size use could have potential implications in both access to targeted therapies and promising innovative drugs on the horizon via enrollment in clinical trials.

Therefore, to investigate the real-world use of different panel size genomic tests and the relationship between area-level socioeconomic deprivation and urban/rural location with genomic test panel use and clinical trial enrollment, we assessed real-world evidence from a single academic center setting. We analyzed by panel size (i) the trends of volume in genomic testing and turnaround times (days between test order date and result date), (ii) the association of genomic test panel size adoption with area-level socioeconomic deprivation and urban/rural location, and (iii) the association between genomic tests, area-level socioeconomic deprivation and urban/rural location with enrollment in clinical trials.

## Materials and Methods

This was a retrospective analysis of electronic health records in the Mayo Clinic Health System. Patients were eligible to be included in the study if they received care within the Mayo Clinic Health System with research authorization, received genomic tests from January 2016 to June 2020, and were older than 18 years. All tumor blocks were pathologically confirmed to have sufficient tissue and eligible tumor content prior to being sent out for genomic testing. Genomic tests included both Mayo Clinic internal tests (FISH, single-gene tests, and multiple gene panels) as well as commercial panels from Foundation Medicine. Clinical data related to patient demographics, cancer diagnoses, and clinical trial enrollment were retrieved from structured and unstructured data sources of institutional collections, including cancer registry, clinical data warehouse, and clinical notes. Patients’ primary cancer types were determined using (i) submitted diagnoses from genomic test reports, and (ii) International Classification of Diseases (ICD) codes from clinical data warehouses. If the cancer type from the submitted diagnosis from the genomic test report contradicted the ICD codes from the clinical data warehouses, we considered the submitted diagnosis from the genomic test reports as the primary cancer type. In total, our cohort included 9,886 eligible subjects and tests (for cases who have had multiple tests, we considered only the earliest test). The study was approved by Mayo Clinic Institutional Review Board (IRB) 13-009317.

### Evaluation of Health Care Disparity for Genomic Tests Utilization

We categorized patients into three categories based on the earliest test type each patient received: single-gene, medium panel (2–49 genes), and large panel (50+ genes). Categorization of medium and large panels was based on common procedural terminology codes used for reimbursement of genomic panel tests, 81445 and 81455, which are defined by whether the test contains >50 genes. Area-level socioeconomic deprivation of each patient was represented by the Area Deprivation Index (ADI), which was composed of 17 indicators reflecting a diverse set of socioeconomic variables including neighborhood-level measures of education, employment, housing quality, and poverty. ADI has been shown to be a good indicator of SES (18). We split the ADI measure into quintiles, with higher values representing greater socioeconomic deprivation (19). We first retrieved patients’ Federal Information Processing System (FIPS) codes by querying patients’ addresses on U.S. Census Bureau website (https://geocoding.geo.census.gov/), which were then linked to the ADI datasets (https://www.neighborhoodatlas.medicine.wisc.edu/; ref. 19), which include ADI national and state ranking for each census block group. In our analysis, ADI state rankings were grouped into three buckets: low area depravity (ADI = 1–3), medium area depravity (ADI = 4–6), and high area depravity (ADI = 7–10). FIPS codes were also used to classify addresses into rural and urban categories based on the Office of Management and Budget metro and nonmetro categories (https://www.ers.usda.gov/data-products/rural-urban-continuum-codes/).

### Evaluation of Utility of Genomic Tests for Clinical Trial Matching

We stratified patients into three categories based on the earliest test type each patient received as described previously. We retrieved patients’ clinical trial accrual status from Mayo's Participant Tracking System (PTrax), an institutional research software program that facilitates management of clinical trial participants’ consent and trial enrollment status and has been used in previous research to study trial participation in real-world settings (20). We considered all patients that were documented in the PTrax system, regardless of future consent denial or screening failure, to have been matched for clinical trial enrollment consideration. Positive clinical trial matching was restricted to those occurring anytime following the genomic test result dates. Associations between ADI and successful clinical trial matching were examined on the basis of the stratified population.

### Statistical Methods

R 4.1.0 was used to analyze statistical significance of difference in turnaround time (Wilcoxon rank-sum tests) and time trend for panel tests (Spearman rank-order correlation test). Turnaround time was defined as days between test order date and result date. We used logistic regression to analyze (i) the relationship between ADI and patients’ adoption of genomic test types and (ii) the relationship between ADI and clinical trial matching stratified by genomic test panel size. Variables in the logistic regression models included patient's gender, age, race (White vs. other), ADI group, urban/rural, cancer stage, lung cancer (yes/no). Given the frequency of use of genomic testing in lung cancer, we stratified both analyses based on cancer type (lung cancer vs. non-lung cancer). To assess the robustness of our results and the influence of the availability of disease stage information, we conducted a sensitivity analysis limiting the analyses to those with known stage of disease.

### Ethics Approval and Consent to Participate

All of the participants provided written informed consent to participate in this study. All protocols were approved by the Mayo Clinic IRB. Mayo Clinic IRB number 20-001474 and 15-003408. All methods were carried out in accordance with the U.S. Common Rule.

### Data Availability Statement

The data used in this study cannot be shared publicly because of the patient health information included in the texts. Please contact the corresponding author for future data access.

## Results

### Study Sample and Patient Characteristics

The study sample included a total of 9,886 patients with cancer. Demographic characteristics of patients are shown in Table 1. Approximately 50% of the patients were female, majority were White (82.9%), and lung cancer was the most common cancer type (22.5%). The majority of the patients in our database had at least some address information available (n = 9,119, 92%) Out of those with a known address, 98% are from the United States, with 49 out of 50 states represented. The top five states were Minnesota (37% of those with known address), Florida (11%), Wisconsin (9%), Iowa (9%), and Arizona (6%). Patients using large panels had a greater proportion of patients from more affluent areas (large panel: 30.6%, medium panel: 16.0%, single gene test: 17.1%), and urban areas (large panel: 55.2%, medium panel: 39.2%, single-gene test: 43.6%) compared with patients taking other two types of tests.

**TABLE 1:** Demographic characteristics of patients

	Large (*N* = 2,909)	Medium (*N* = 2,384)	Single-gene (*N* = 4,593)	Total (*N* = 9,886)
Race				
White	2,478 (85.2%)	2,002 (84.0%)	3,713 (80.8%)	8,193 (82.9%)
Asian/Pacific Islander	86 (3.0%)	38 (1.6%)	76 (1.7%)	200 (2.0%)
African American	91 (3.1%)	16 (0.7%)	61 (1.3%)	168 (1.7%)
Hispanic/Latino	98 (3.4%)	30 (1.3%)	105 (2.3%)	233 (2.4%)
Other (including American Indian)	64 (2.2%)	40 (1.7%)	86 (1.9%)	190 (1.9%)
Unknown	92 (3.2%)	258 (10.8%)	552 (12.0%)	902 (9.1%)
Gender				
Female	1,465 (51.0%)	974 (44.9%)	2,110 (51.4%)	4,549 (49.7%)
Male	1,405 (49.0%)	1,194 (55.1%)	1,997 (48.6%)	4,596 (50.3%)
Missing	39	216	486	741
Age				
Missing	39	216	486	741
Mean (SD)	61.29 (14.18)	66.08 (12.23)	62.03 (14.51)	62.76 (14.02)
Median (Q1, Q3)	63.00 (54.00, 71.00)	68.00 (58.00, 75.00)	64.00 (53.00, 72.00)	64.00 (55.00, 73.00)
Range	18.00–94.00	18.00–94.00	18.00–98.00	18.00–98.00
Test.year				
2016	319 (11.0%)	234 (9.8%)	793 (17.3%)	1346 (13.6%)
2017	740 (25.4%)	560 (23.5%)	487 (10.6%)	1787 (18.1%)
2018	701 (24.1%)	611 (25.6%)	1178 (25.6%)	2490 (25.2%)
2019	985 (33.9%)	668 (28.0%)	1468 (32.0%)	3121 (31.6%)
2020	164 (5.6%)	311 (13.0%)	667 (14.5%)	1142 (11.6%)
Area deprivation index				
Low (values 1–3)	890 (30.6%)	381 (16.0%)	785 (17.1%)	2,056 (20.8%)
Medium (values 4–6)	561 (19.3%)	438 (18.4%)	742 (16.2%)	1,741 (17.6%)
High (values 7–10)	404 (13.9%)	445 (18.7%)	772 (16.8%)	1,621 (16.4%)
Unknown	1,054 (36.2%)	1,120 (47.0%)	2,294 (49.9%)	4,468 (45.2%)
Cancer type				
Lung	390 (13.4%)	1,294 (54.3%)	541 (11.8%)	2,225 (22.5%)
Colorectal	208 (7.2%)	297 (12.5%)	765 (16.7%)	1,270 (12.8%)
Breast	132 (4.5%)	1 (%)	736 (16%)	869 (8.8%)
Other gastrointestinal	228 (7.8%)	179 (7.5%)	351 (7.6%)	758 (7.7%)
Pancreas	171 (5.9%)	5 (0.2%)	487 (10.6%)	663 (6.7%)
Melanoma	73 (2.5%)	365 (15.3%)	214 (4.7%)	652 (6.6%)
Soft tissue	125 (4.3%)	26 (1.1%)	253 (5.5%)	404 (4.1%)
Gynecologic	291 (10%)	1 (%)	53 (1.2%)	345 (3.5%)
Brain	185 (6.4%)	11 (0.5%)	107 (2.3%)	303 (3.1%)
Prostate	115 (4%)	0 (%)	33 (0.7%)	148 (1.5%)
Thyroid	100 (3.4%)	1 (%)	29 (0.6%)	130 (1.3%)
Bladder	78 (2.7%)	1 (%)	28 (0.6%)	107 (1.1%)
Other	608 (20.9%)	116 (4.9%)	369 (8%)	1,093 (11.1%)
Unknown	205 (7%)	87 (3.6%)	627 (13.7%)	919 (9.3%)
Stage				
Localized	554 (19.0%)	464 (19.5%)	1,191 (25.9%)	2,209 (22.3%)
Advanced/Metastatic	1,395 (48.0%)	1,062 (44.5%)	636 (13.8%)	3,093 (31.3%)
Unknown	960 (33.0%)	858 (36.0%)	2,766 (60.2%)	4,584 (46.4%)
Urban/Rural				
Urban	1,605 (55.2%)	935 (39.2%)	2,004 (43.6%)	4,544 (46.0%)
Rural	476 (16.4%)	728 (30.5%)	1,109 (24.1%)	2,313 (23.4%)
Unknown	828 (28.5%)	721 (30.2%)	1,480 (32.2%)	3,029 (30.6%)

### Real-world Trends in Genomic Testing

**FIGURE 1:**
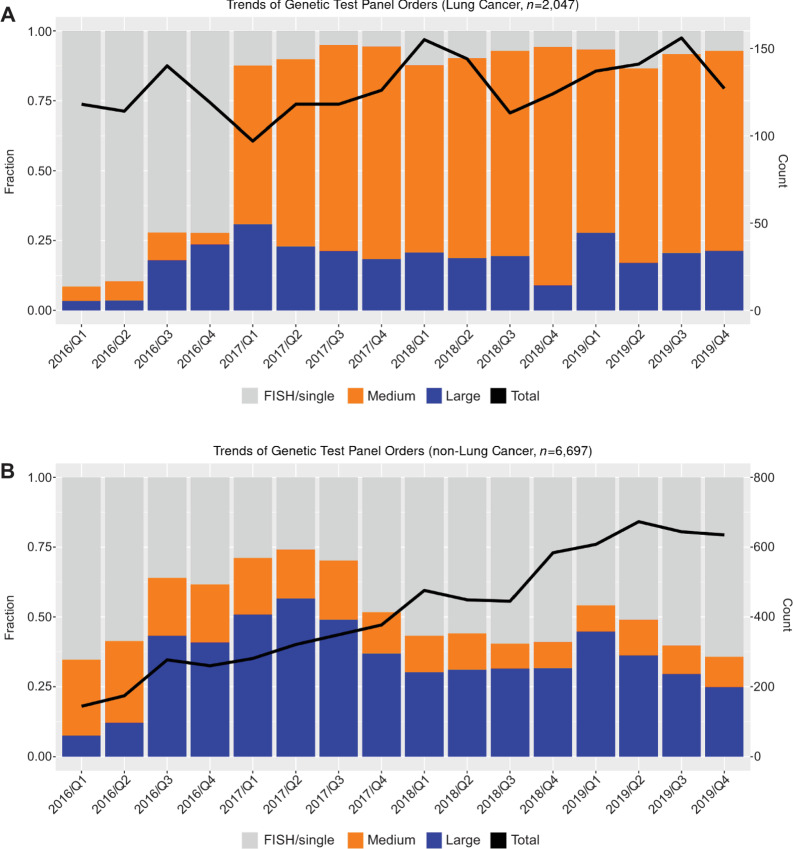
Trends of single-gene, medium, large genomic test panel distribution among patients with lung cancer and non-lung cancer. **A,** Proportion and total number of genetic tests over time by size [large panel (50+ genes), medium panel (2 to 49 genes), single-gene] among patients with a lung cancer diagnosis. **B,** Proportion and total number of genetic tests over time by size [large panel (50+ genes), medium panel (2 to 49 genes), single-gene] among patients with a non-lung cancer diagnosis.

Overall, an increase in use of genomic testing was observed over time (Fig. 1); however, the use of genomic testing differed by tumor type, with a greater increase in the rate of testing among patients with non-lung cancer. In addition, differences in genomic panel size adoption between patients with lung cancer and non-lung cancer were observed. Among patients with lung cancer, the fraction of single-gene panel orders experienced a reduction (r = −0.591, *P* < 0.05), while medium panel orders had the greatest increase since 2017 (*r* = 0.635, *P* < 0.05). In contrast, among patients with non-lung cancer, the proportion of medium panel orders had a significant decrease (*r* = −0.874, *P* < 0.05), while the fraction of single-gene panel orders experienced an increase (*r* = 0.335, *P* = 0.204).

The mean turnaround time for single-gene tests (9.1 days) was significantly lower than the medium (17.6 days) and large (16.2 days) panels (*P* < 0.05; Table 2). The proportion of patients whose test results returned greater than 28 days later was 2.8% (single-gene), 5.3% (medium panel), and 6.2% (large panel), respectively.

**TABLE 2:** Time to test return (turnaround time) for large panel, medium panel, and single-gene genomic tests

	Median (IQR)	Mean	>28 days turnaround (%)
Large	13 (11, 16)	16.2	6.2%
Medium	16 (14, 20)	17.6	5.3%
Single-gene	7 (4, 11)	9.1	2.8%

### Association of Area-level Deprivation and Rurality with Genomic Test Type

Lower ADI (more affluent areas) was found to be associated with a greater use of medium/large panels compared with single-gene tests (Table 3). Among all cancer types, the most area-deprived group (ADI = 7–10) had a 29% lower odds [OR (95% CI): 0.71 (0.61–0.83), *P* < 0.01] of receiving a genomic test panel (medium or large) compared with patients in the most affluent areas (ADI = 1–3). The gap was even wider for large versus single-gene and large versus medium panels comparisons as patients living in the areas with the highest area deprivation had a 42% [OR (95% CI): 0.58 (0.49–0.69), *P* < 0.01] and 46% [OR (95% CI): 0.54 (0.45–0.66), *P* < 0.01] lower odds of receiving a large panel genomic test. Results differed when split by patients with lung cancer versus non-lung cancer. While all patients with non-lung cancer in medium and high area deprivation locations were less likely to receive a panel test or large panel test, there was no significant difference in receipt of panel size among patients with lung cancer except for those living in the locations with the highest area deprivation having lower odds of receiving a large panel versus a medium panel test [OR (95% CI): 0.60 (0.41–0.86), *P* = 0.006].

**TABLE 3:** Association of panel size with ADI and urban/rural status^a^

Tests for all cancer types (*N* = 9,886)						
	Medium+Large vs. Single-gene (*n* = 9,886)		Large vs. Single-gene (*n* = 7,502)		Large vs. Medium (*n* = 5,293)	
Value	OR	*P*-value	OR	*P*-value	OR	*P*-value
Area deprivation index						
Low area depravity (values 1–3)	Ref.	—	Ref.	—	Ref.	—
Medium area depravity (values 4–6)	0.86 (0.74–0.99)	*P* = 0.035	0.75 (0.64–0.89)	*P* = 0.001	0.69 (0.57–0.83)	*P* < 0.001
High area depravity (values 7–10)	0.71 (0.61–0.83)	*P* < 0.001	0.58 (0.49–0.69)	*P* < 0.001	0.54 (0.45–0.66)	*P* < 0.001
Rural/Urban						
Urban	Ref.	—	Ref.	—	Ref.	—
Rural	0.85 (0.76–0.96)	*P* = 0.006	0.54 (0.47–0.63)	*P* < 0.001	0.42 (0.36–0.48)	*P* < 0.001
**Tests for lung cancer only (*N* = 2,231)**						
	**Medium+Large vs. Single-gene** **(*n* = 2,231)**		**Large vs. Single-gene** **(*n* = 937)**		**Large vs. Medium** **(*n* = 1684)**	
**Value**	**OR**	***P*-value**	**OR**	***P*-value**	**OR**	***P*-value**
Area deprivation index						
Low area depravity (values 1–3)	Ref.	—	Ref.	—	Ref.	—
Medium area depravity (values 4–6)	0.90 (0.64–1.26)	*P* = 0.536	0.75 (0.49–1.15)	*P* = 0.187	0.77 (0.54–1.09)	*P* = 0.134
High area depravity (values 7–10)	1.00 (0.71–1.41)	*P* = 0.989	0.76 (0.48–1.20)	*P* = 0.240	0.60 (0.41–0.86)	*P* = 0.006
Rural/Urban						
Urban	Ref.	—	Ref.	—	Ref.	—
Rural	0.93 (0.73–1.21)	*P* = 0.638	0.44 (0.30–0.65)	*P* < 0.001	0.38 (0.27–0.53)	*P* < 0.001
**Tests for non-lung cancer only (*N* = 7,655)**						
	**Medium+Large vs. Single-gene** **(*n* = 7,655)**		**Large vs. Single-gene** **(*n* = 6,565)**		**Large vs. Medium** **(*n* = 3,609)**	
**Value**	**OR**	***P*-value**	**OR**	***P*-value**	**OR**	***P*-value**
Area deprivation index						
Low area depravity (values 1–3)	Ref.	—	Ref.	—	Ref.	—
Medium area depravity (values 4–6)	0.84 (0.72–0.99)	*P* = 0.035	0.76 (0.64–0.91)	*P* = 0.003	0.68 (0.53–0.87)	*P* = 0.002
High area depravity (values 7–10)	0.63 (0.53–0.75)	*P* < 0.001	0.56 (0.46–0.68)	*P* < 0.001	0.62 (0.47–0.82)	*P* = 0.001
Rural/Urban						
Urban	Ref.	—	Ref.	—	Ref.	—
Rural	0.80 (0.70–0.91)	*P* = 0.001	0.55 (0.47–0.65)	*P* < 0.001	0.38 (0.31–0.47)	*P* < 0.001

^a^Model included covariates for gender, age, ADI group, urban/rural, race, stage, lung cancer.

Patients living in rural areas were also found to be associated with a lower use of medium/large panels compared to single-gene tests, as well as a lower use or large panels compared with single-gene test and medium-sized panels. In contrast to the ADI results, when split by lung cancer versus non-lung cancer, results were similar with the exception of rural patients with lung cancer not having a significant difference in odds of receiving any sized panel test versus a single-gene test. Results were directionally similar when examining the subgroup of patients with known stage of disease (Supplementary Table S1).

### Association of Area-level Deprivation with Genomic Test Type and Clinical Trial Matching

Approximately 1 in 5 (19.9%) of the patients taking genomic tests were matched to an eligible clinical trial. Compared with patients living in the most affluent areas, patients living in locations with the highest area deprivation had lower odds of being matched to a clinical trial if receiving a medium panel test [OR (95% CI): 0.69 (0.49–0.97), *P* = 0.03] (Table 4). In contrast, the odds of being matched to a clinical trial was not significantly different between patients living in the lowest and highest area deprivation locations and receiving a large panel test [OR (95% CI): 1.04 (0.64–1.67), *P* = 0.86]. Rural patients receiving large panel tests also had a higher odds of being matched to a clinical trial [OR (95% CI): 1.76 (1.21–2.55), *P* < 0.01] while there was no difference in odds of trial matching for rural patients receiving medium-sized panels or single-gene tests (*P* > 0.05). Results were directionally similar when examining the subgroup of patients with known stage of disease (Supplementary Table S2).

**TABLE 4:** Association of area deprivation index and urban/rural status with clinical trial matching (success vs. failed)^a^

	Large (*N* = 2,909)		Medium (*N* = 2,384)		Single-gene (*N* = 4,593)	
Value	OR	*P*-value	OR	*P*-value	OR	*P*-value
Area deprivation index						
Low area depravity (values 1–3)	Ref.	—	Ref.	—	Ref.	—
Medium area depravity (values 4–6)	0.98 (0.63–1.51)	*P* = 0.935	0.88 (0.63–1.22)	*P* = 0.448	0.87 (0.68–1.10)	*P* = 0.234
High area depravity (values 7–10)	1.04 (0.64–1.67)	*P* = 0.858	0.69 (0.49–0.97)	*P* = 0.033	0.84 (0.66–1.08)	*P* = 0.174
Rural/Urban						
Urban	Ref.	—	Ref.	—	Ref.	—
Rural	1.76 (1.21–2.55)	*P* < 0.001	1.09 (0.85–1.39)	*P* = 0.502	0.97 (0.80–1.17)	*P* = 0.765

^a^Model included covariates for gender, age, ADI group, urban/rural, race, stage, lung cancer.

## Discussion

In our work, we utilized an institutional oncology cohort with diverse cancer types to examine (i) genomic test utilization trend and turnaround time for genomic test panels of three different sizes, (ii) the relationship between sizes of genomic panels used by patients with ADI and urban/rural location, and (iii) the relationship between clinical trial matching with ADI and urban/rural location by sizes of genomic panels used by patients.

Our study found an increasing trend of using broader size panels over the years. In patients with lung cancer starting in 2017, there was a substantial increase in orders for medium panels while orders for single-gene tests significantly declined. For patients with non-lung cancer, an increasing trend in all three types of tests was observed. This distinct trend pattern between patients with lung cancer and non-lung cancer may be due to the development and adoption of lung cancer–specific medium panels, which assessed multiple guideline-approved gene targets simultaneously in EGFR, BRAF, KRAS, HRAS, NRAS, ALK, ERBB2, MET, ALK, ROS1, RET, and NTRK1 genes. Meanwhile in patients with non-lung cancer, an increasing trend in all three types of panels was observed. Recent advances in targeted therapeutics for patients with lung cancer might explain the distinct trend pattern of molecular testing utilization across tumor types. For the patients with lung cancer, medium panels, comprehensively includes all gene targets with available drugs that are approved by regulatory agencies. On the other hand, standard options for tumor NGS informed treatment in patients with other tumor types is limited, possibly leading to larger panel testing to identify a target for off-label treatment or clinical trial referral. In addition, estimation of tumor mutation burden (TMB) as well as detection of mismatch repair deficiency is often relevant in tumors other than lung: a high TMB (≥10) or classification of a tumor as mismatch repair deficient, can justify the use of immune checkpoint inhibitors that might otherwise not be indicated. Conversely, immune checkpoint inhibitors are indicated in various settings of lung cancer regardless of TMB whereas these tumors are universally mismatch repair proficient.

Another consideration for the choice of test size may be turnaround time. In this analysis, single-gene panels had significantly shorter turnaround time compared with large- and medium-sized panel tests. The proportion of tests results returning >28 days was low across all three test sizes (<∼6%), although, this occurred less with the single-gene tests (∼3%). Despite small, the number of cases where it takes more than 28 days to obtain molecular information is clinically significant when considering individual patients. While long turnaround times were infrequent, this may be an important consideration for select patients, especially for patients whose diseases are progressing rapidly and need immediate treatment. On the other hand, for patients on existing treatment and seeking alternative options or for those who could tolerate a longer turnaround time, taking a broader panel test might bring more treatment possibilities, including trial participation opportunities, while minimizing risking. Therefore, careful consideration to the individual patient needs should be given when determining the optimal test size and associated turnaround times.

SES and rural/urban location are additional potential factors which could influence the utilization trends. Previous studies (21) have found that SES to be associated with use of genomic tests, while others have found biomarker testing in rural areas to be suboptimal or lower than in urban areas (22, 23). In our study, we found that patients living in more socioeconomically deprived areas or rural areas to be less likely to receive large- or medium-sized panel tests. One potential reason for this may be the differences in coverage and insurance types among the different ADI cohorts and between the rural and urban cohorts. For example, those living in the most socioeconomically disadvantaged areas may be more likely to have Medicaid (24), which may be less likely to have coverage policies for comprehensive biomarker testing (25). Meanwhile, lack of insurance coverage has been cited as a common barrier to testing in rural areas (22). In addition, an analysis of coverage decisions for MGPT among commercial payers has shown large variability among payers, with many coverage restrictions based on panel size (26). Given insurance data were not available in our dataset, further research examining the association between coverage, ADI, and panel size utilization would be needed to confirm these hypotheses.

Given the lower use of large panel tests by patients in located in high-level socioeconomic deprivation and rural areas, we explored whether this may result in an association with clinical trial participation. Previous studies have suggested an association with the use of NGS and trial participation (27, 28); however, lower SES and rural areas have also been linked to lower trial participation (29). Transportation/travel distance and cost, among other study burden barriers have been cited as reasons for challenges with recruitment and may particularly impact lower SES and rural patients (30).

Here, we found that in the medium panel cohorts, high area-level socioeconomic deprivation was associated with lower odds of being considered for a trial. However, the relationship did not hold true for large panel cohort, where there was no association found between ADI and trial consideration. There are several potential explanations for this observation. First, the results potentially suggest that large panels may have a health equity impact due to the large number of genes covered, thus creating more equal opportunities to be matched to a clinical trial for all patients using these tests. If this is the case, access to large panels may be considered a barrier to clinical trial matching and greater efforts should be made to widen their access to all patients. Second, it is possible that it is the lack of clinical trial matching in patients with higher SES rather than more frequent matching for patients of lower SES that drives this trend. Taking patients with lung cancer for example, patients with lung cancer and higher SES tend to have healthier lifestyles, including lower consumption of tobacco products that is usually associated with specific target oncogenes that match to relevant precision oncology clinical trials. A notable exception is KRAS G12C, a smoking-related mutation with clinical trial options only in the past 2 years and an FDA approval in 2021. It is possible that some of the trends observed in the study herein might change to reflect a rapidly evolving dynamic field. Alternatively, patients taking large panel tests with high SES might have already gone through several lines of therapy and were only looking to expand their treatment options rather than seeking to be matched to a trial.

In contrast to the ADI findings, we found rural patients receiving large panel tests to be associated with almost two times greater odds of being considered for a clinical trial; however, there was no association with rurality and trial consideration among patients receiving medium panel or single-gene tests. The reasons for this are unknown, however may be related to the higher participation of rural patients in clinical trials at this multicenter academic institution which has been reported previously (20). The results suggest that potentially improving access to large panel tests for rural patients may further increase trial consideration and efforts to address barriers to access of large panels may be warranted. The findings here however should be interpreted with caution and may be impacted by unmeasured factors which were unavailable in our data, such as imbalances in the line of therapy between ADI cohort. Given trial matching is a complicated process and our work only provides a simplified view of how ADI, geographic location, and genomic test panel size were associated with trial consideration, these findings should be considered exploratory, with additional research needed to further explore the potential association.

The disparities identified in this study have a number of implications for achieving equitable cancer care. One potential solution to narrowing disparities may be provider education. Previous research has shown provider education to improve testing rates and targeting education at providers who care for low-income patients may help improve access to testing for those living in high area depravity locations (31). Another opportunity to improve testing in patients living in high deprivation or rural areas may be through facilitating state Medicaid coverage. Patients in these areas have been linked to a higher likelihood of using Medicaid and ongoing state legislative efforts to facilitate access to testing may help remove a key access barrier (32–34). Improving access to biomarker testing in this population has important clinical implications as well, where lack of testing has been associated with poorer survival (15).

Our study has several limitations: (i) our study did not include line of therapy, cancer stage, or insurance type as covariates in our regression analysis as they were not fully available to us at the time of analysis; however, these are important factors when considering genomic test panels and clinical trial enrollment. Future research is needed to incorporate these factors into analyses and understand any role they may play in disparities in use of different genomic panel sizes. (ii) We utilized real-world data in our study, which might suffer from missingness, for example, undocumented clinical trial enrollment information or tumor staging. (iii) Our cohort only includes patients taking Foundation Medicine test and Mayo Internal comprehensive solid tumor panel test as large panel cohort. However, there are patients taking large panels from other vendors such as Tempus.

In conclusion, in an institutional oncology cohort, there was an increase in panel tests over time. Patients in high area-level socioeconomic deprivation, however, were less likely to use large panel tests and be considered for clinical trials if using medium-sized panel tests. Patients in rural areas were also less likely to receive large panel tests, however, were more likely to be considered for clinical trials if using a large panel, suggesting improving access to large panel tests may be warranted. Further research is needed to explore the potential association between large panel tests, area-level socioeconomic deprivation, geographic location, and clinical trial consideration.

## Supplementary Material

Supplementary Table S1 Supplemental Table S1 shows the association of area level deprivation and rurality with genomic test type in the subgroup of patients with known stage of disease

Supplementary Table S2 Supplementary Table S2 shows the association of area level deprivation and rurality with clinical trial matching among patients with known stage of disease
