# Supplementary material for: Real-world Trends, Rural-urban Differences, and Socioeconomic Disparities in Utilization of Narrow versus Broad Next-generation Sequencing Panels
Source: Cancer Res Commun. 2024 Feb 5;4(2):303–11. doi: 10.1158/2767-9764.CRC-23-0190 (PMC10840454; doi:10.1158/2767-9764.CRC-23-0190)
Supplement: Supplementary Table S1 — Supplemental Table S1 shows the association of area level deprivation and rurality with genomic test type in the subgroup of patients with known stage of disease [file crc-23-0190-s01.docx]

**Supplementary Table S1** Association of panel size with Area Deprivation Index and Urban/Rural status Among Patients with Known Disease Stage*

| Tests for All Cancer Types (N=5,302) | | | | | | |
| --- | --- | --- | --- | --- | --- | --- |
| Value | Medium+Large vs. Single-gene  (n=5,302) | | Large vs. Single-gene  (n=3,776) | | Large vs. Medium  (n=3,475) | |
|  | **OR** | **p-value** | **OR** | **p-value** | **OR** | **p-value** |
| Area Deprivation Index |  |  |  |  |  |  |
| Low Area Depravity  (values 1-3) | Ref. | - | Ref. | - | Ref. | - |
| Medium Area Depravity  (values 4-6) | 0.94  (0.78-1.14) | p=0.551 | 0.79  (0.64-0.99) | p=0.037 | 0.69  (0.55-0.85) | p=0.001 |
| High Area Depravity  (values 7-10) | 0.85  (0.69-1.04) | p=0.120 | 0.71  (0.56-0.90) | p=0.004 | 0.57  (0.45-0.72) | p<0.001 |
| Rural/Urban |  |  |  |  |  |  |
| Urban | Ref. |  | Ref. |  | Ref. |  |
| Rural | 0.82  (0.70-0.96) | p=0.014 | 0.54  (0.45-0.66) | p<0.001 | 0.45  (0.37-0.54) | p<0.001 |
| Tests for Lung Cancer Only (N=1,780) | | | | | | |
| Value | Medium+Large vs. Single-gene (n=1,780) | | Large vs. Single-gene  (n=734) | | Large vs. Medium  (n=1,409) | |
|  | **OR** | **p-value** | **OR** | **p-value** | **OR** | **p-value** |
| Area Deprivation Index |  |  |  |  |  |  |
| Low Area Depravity  (values 1-3) | Ref. | - | Ref. | - | Ref. | - |
| Medium Area Depravity  (values 4-6) | 0.93  (0.64-1.35) | p=0.713 | 0.77  (0.49-1.22) | p=0.267 | 0.75  (0.52-1.08) | p=0.126 |
| High Area Depravity  (values 7-10) | 1.06  (0.72-1.56) | p=0.758 | 0.76  (0.46-1.25) | p=0.279 | 0.56  (0.38-0.83) | p=0.004 |
| Rural/Urban |  |  |  |  |  |  |
| Urban | Ref. |  | Ref. |  | Ref. |  |
| Rural | 0.85  (0.64–1.12) | p=0.250 | 0.36  (0.23–0.54) | p<0.001 | 0.33  (0.23–0.47) | p<0.001 |
| Tests for Non-Lung Cancer Only (N=3,522) | | | | | | |
| Value | Medium+Large vs. Single-gene  (n=3,522) | | Large vs. Single-gene  (n=3,042) | | Large vs. Medium  (n=2,066) | |
|  | **OR** | **p-value** | **OR** | **p-value** | **OR** | **p-value** |
| Area Deprivation Index |  |  |  |  |  |  |
| Low Area Depravity  (values 1-3) | Ref. | **-** | Ref. | **-** | Ref. | - |
| Medium Area Depravity  (values 4-6) | 0.94  (0.74-1.18) | p=0.585 | 0.82  (0.64-1.05) | p=0.118 | 0.68  (0.48-0.95) | p=0.025 |
| High Area Depravity  (values 7-10) | 0.77  (0.60-0.99) | p=0.040 | 0.71  (0.54-0.93) | p=0.013 | 0.73  (0.50-1.07) | p=0.106 |
| Rural/Urban |  |  |  |  |  |  |
| Urban | Ref. |  | Ref. |  | Ref. |  |
| Rural | 0.74  (0.61-0.91) | P=0.004 | 0.59  (0.47-0.74) | p<0.001 | 0.45  (0.34-0.60) | p<0.001 |

*Model included covariates for gender, age, ADI group, urban/rural, race, stage, lung cancer
