# Supplementary material for: Real-world Trends, Rural-urban Differences, and Socioeconomic Disparities in Utilization of Narrow versus Broad Next-generation Sequencing Panels
Source: Cancer Res Commun. 2024 Feb 5;4(2):303–11. doi: 10.1158/2767-9764.CRC-23-0190 (PMC10840454; doi:10.1158/2767-9764.CRC-23-0190)
Supplement: Supplementary Table S2 — shows the association of area level deprivation and rurality with clinical trial matching among patients with known stage of disease [file crc-23-0190-s02.docx]

**Supplementary Table S2** Association of Area Deprivation Index and Urban/Rural Status with Clinical Trial Matching (Success vs Failed)* Among Patients with Known Disease Stage.

| Value | Large (N=1,949) | | Medium (N=1,526) | | Single-gene (N=1,827) | |
| --- | --- | --- | --- | --- | --- | --- |
|  | **OR** | **p-value** | **OR** | **p-value** | **OR** | **p-value** |
| Area Deprivation Index |  |  |  |  |  |  |
| Low Area Depravity  (values 1-3) | Ref. | - | Ref. | **-** | Ref. | **-** |
| Medium Area Depravity  (values 4-6) | 0.70  (0.37-1.29) | p=0.265 | 0.88  (0.58-1.32) | p=0.525 | 1.15  (0.83-1.59) | p=0.402 |
| High Area Depravity  (values 7-10) | 0.88  (0.46-1.64) | p=0.690 | 0.76  (0.49-1.16) | p=0.197 | 1.05  (0.75-1.47) | p=0.791 |
| Rural/Urban |  |  |  |  |  |  |
| Urban | Ref. |  | Ref. |  | Ref. |  |
| Rural | 1.29  (0.76-2.17) | p=0.343 | 1.09  (0.80-1.48) | p=0.576 | 0.84  (0.65-1.09) | p=0.189 |

*Model included covariates for gender, age, ADI group, urban/rural, race, stage, lung cancer
